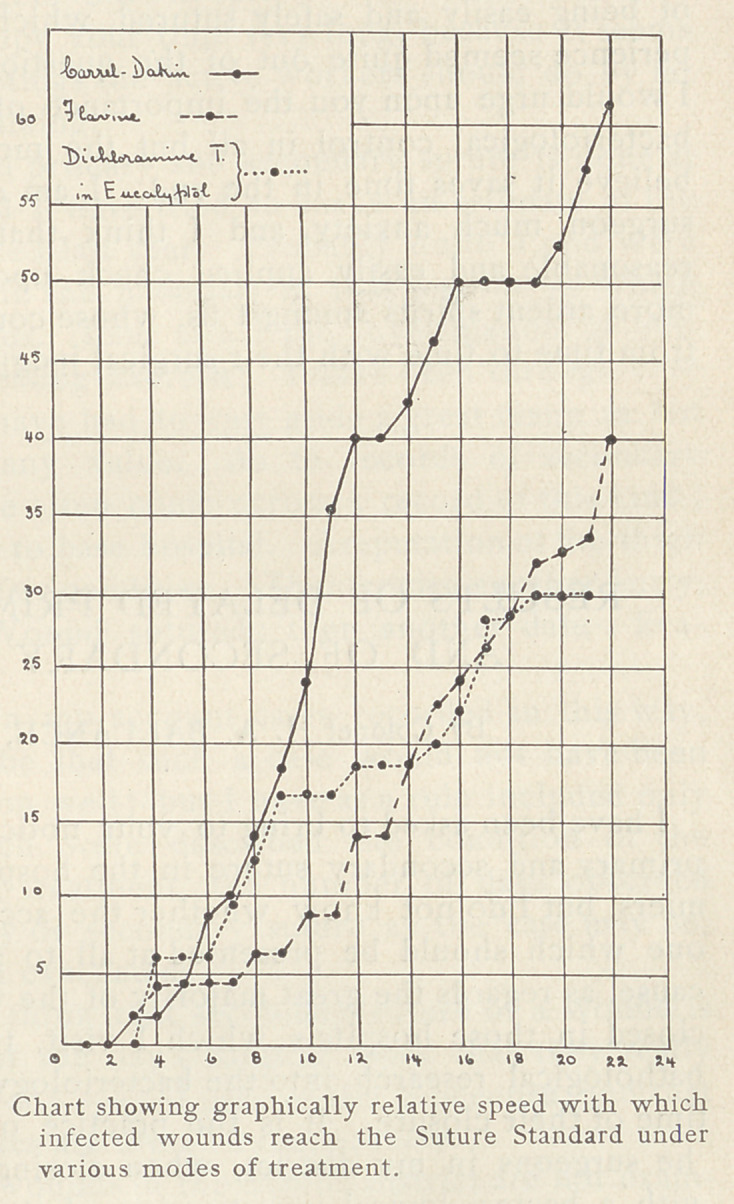# Suture of War Wounds at Base Hospitals

**Published:** 1918-03

**Authors:** John T. Morrison


### SUTURE OF WAR WOUNDS AT BASE HOSPITALS

BY

JOHN T. MORRISON, F. R. C. S., Temp. Capt., R. A. M. C.

I have been asked to speak this afternoon of closure of wounds at the Base and to this subject I intend closely to adhere.

We have heard today already something of the brilliant work which has been done by our colleagues in the field of primary suture, and, after the wonderful series of statistics put forward by Dr. Lemaître, I feel that my own small series is not of much value to offer. It may, however, be of some interest, insofar as the point of view is different.

The question of closing wounds at the Base, so far as one can see, will always be with us. There will always, I fear, be some cases where, for military or perhaps anatomical reasons, primary suture is inadvisable or impossible. There will, too, be a percentage — no doubt a diminishing percentage — in which success has not been attained. These must be dealt with by delayed primary or by secondary suture.

By the term " delayed primary suture " we mean cases of suture performed within the first five days after being hit, exclusive, of course, of cases sutured primarily. It is intended to exclude those cases in which by the full development of the natural resisting forces of the body by active immunization an infection has been overcome. It includes on the other hand those wounds left open not so much for the treatment of sepsis as to make reasonably certain that a serious infection is not going to develop.

On the advantages I need not dwell. They may be regarded from two points of view, the military and the surgical. To the military man they appeal by reason of increased economy of time, material, and personnel effected by the shortening of the period of recovery. The surgeon is attracted by the rapid restoration practically to the normal and improved functional results.

I am able to put before you the results of some two hundred cases of wound suture, and of these, almost one-third were treated by my colleague Capt. Hartley, R. A. M. C., to whom I am indebted for permission to refer to them. Of the 192 cases, 46 were cases of delayed primary suture. During the last six months we have been able to perform delayed primary suture in over 10 o/o of our admissions. In view of the fact that these are cases collected during the ordinary course of evacuation, without any special arrangement for suitable cases being made — in view of the fact that the cases primarily sutured elsewhere have to be deducted and that the cases coming into our hands, all of them serious, have been operated on by many different surgeons, of varying degrees of skill, I think that the ratio of 10 o/o arriving in a condition fit for immediate suture, is one very creditable to the work done in second line units. The proportion I am glad to say is constantly growing.

I have gone into the figures relating to these cases and I will not burden you with them all. There is only one fact which has stood out clear and incontestable, and that is the value of what I may call the " Suture Standard " as an indication for suture. To this I will refer in detail later. All the cases appeared clinically suitable for closure.

*Delayed Primary Suture.*

| | Suture Standard Reached | Suture Standard *Not* Reached |
|---|---|---|
| Total. . . . . . . . . . . . . . . . . . . . | 35 | 11 |
| Primary Union . . . . . . . . . . . . . . . | 80 o/o | 27 o/o |
| Incomplete success . . . . . . . . . . . . | 14 o/o | 46 o/o |
| but Evacuated *Healed* after 24 days and 27 days respectively. | | |
| Evacuated but Unhealed after 24 and 27 days respectively . . . . . . . . . . . . . . . | 6 o/o | 27 o/o |

A glance at the table shows the great difference in the quality of the success obtained when the Suture Standard is carefully observed. In this group of 35 cases 80 o/o healed by first intention and in 14 o/o more, partial success was secured, that is to say there was not accurate apposition of skin edges or else stitches cut through or slight stitch infection may have occurred. In the bulk of these cases it was an actual physical difficulty in closing the wound that accounted for the absence of complete success. Added together they represent to my mind 94 o/o of highly satisfactory results. The remaining 6 o/o were still unhealed when evacuated and in so far may be regarded as failures. Contrast all this with the case of wounds, clinically suitable for suture, examined in the same way and sutured in spite of not being up to suture standard: 27 o/o of primary union instead of 80 o/o. In the list of incomplete successes, 46 o/o in all, the factor was not so much physical difficulty in closure as mild sepsis that was responsible for lack of success. In place of 6 o/o evacuated unhealed we have 27 o/o, and amongst them one case where, on account of serious sepsis, the stitches had to be removed and the patient I believe was definitely injured by the attempt at closure.

These figures I should say include wounds of all kinds excluding, however, perforating injuries of the skull, chest, and abdomen. The relative proportions of wounds of the soft parts, bones, and joints, — the latter of course including many bone injuries, — for the last six months are shown in the accompanying table. The table actually refers to the total admissions and in the series of wounds sutured the percentage of wounds of soft parts only is rather higher, as one would expect, though the difference is not great.

| | | |
|---|---|---|
| *Wounds of soft parts* | | 37 o o |
| *Compound fractures:* | | |
| Humerus | 9 o/o | |
| Radius and ulna | 4.5 o/o | |
| Femur | 6.5 o/o | |
| Tibia and fibula | 11 o/o | |
| Other bones } Parietal fractures } | 15 o/o | |
| | | 46 o/o |
| Shoulder | 4 o/o | |
| Elbow | 2 o/o | |
| Wrist | 2 o/o | |
| Hip | — | |
| Knee | 6.5 o/o | |
| Ankle | 2.5 o/o | |
| | | 17 o/o |
| Total | | 100 o/o |

The indications for delayed primary suture are both clinical and bacteriological, and perhaps I can put the problem most clearly if I run briefly over the method I use in dealing with a patient on his admission to a Base Hospital. With us seriously wounded men usually arrive during the night, from thirty-six hours to three days after being wounded. They are washed and put to bed, made as comfortable as possible, lightly fed and encouraged to sleep. The next morning the men and their wounds are carefully examined and where there is any possibility of a foreign body being still *in situ*, the X-Rays are employed. If the temperature is not rising and the pulse is quite satisfactory, a moderate degree of fever is no contra-indication. When the superficial dressings are removed there must be no trace of spreading inflammation. It should be remembered, however, that a considerable degree of swelling, especially in cases of fracture, may be caused by the trauma alone, and is not necessarily incompatible with a low degree of infection or even with a wound which proves sterile on culture. The deep dressing in such favorable cases I disturb only just enough to enable me to pass a platinum loop into the depths of the wound. The wound surface when so exposed is often covered with a thin, perfectly clear layer of coagulated lymph. The platinum loop passed into the depths is made use of to remove exudate from all the most doubtful corners, not forgetting the wound edges, and from this smears are made. It is from these smears examined according to the Carrel technique, that the decision is made as to whether the wound is or is not up to " Suture Standard ". If not more than one organism, bacillus or coccus, is seen in any one field, or in the case of diplococcal types, not more than one pair in any three consecutive fields, that wound is said to be for the time up to suture standard. Next morning the wound is dressed again the whole wound being exposed, smears are again made in the same way, this second examination being, if possible, even more searching than the first, and if an equally favorable report is given, the wound is immediately closed.

Of the previous treatment and technique of suture, I intend to speak later.

Turning next to the question of secondary suture, we are faced by new problems. We have to deal here with wounds in which an infection has been combated. The wound has become covered with granulation tissue and side by side with this there has developed a power of overcoming bacterial invasion. This of course is a factor in the surgeon's favor. Ranged against him he finds the fact that latent foci of infection are becoming buried in the tissues and that the soft parts are being fixed by cicatrization in abnormal positions, keeping all this in mind he can decide what are the indications and what the con·ra indications for secondary suture.

But first let me put before you the results of 146 cases. Again I have to call your attention to the value of the " Suture Standard ".

*Secondary suture.*

| | Suture Standard Reached | Suture Standard Not reached |
|---|---|---|
| Total | 107 | 37 |
| Primary Union | 64 o/o | 15 o/o |
| Incomplete success. but Evacuated Healed after 23 and 26 days respectively. | 27 o/o | 46 o/o |
| Evacuated *Unhealed* after 23 and 26 days respectively | 9 o/o | 39 o/o |

Of the 107 cases up to suture standard at the time of closing, 64 o/o healed by primary union and, in 27 o/o, complete success was just missed, a total of 91 o/o of satisfactory results. The cases evacuated unhealed numbered 9 o/o. In the other series we have again an altogether different quality of success. Only 15 o/o healed by first intention, 46 o/o falling under the heading " incomplete success " — together amounting to 61 o/o instead of the 91 o/o of the cases up to suture standard. There were again 39 o/o evacuated unhealed instead of only 9 o/o. Amongst these 146 cases there were 5 in which the stitches were removed on account of serious sepsis, 5 that may be regarded as total failures. In three of these the infective agent was a streptococcus, in one a diplococcus and in the fifth the B. pyocyaneus. In these cases as well as in our whole series the cultural results are still too incomplete to allow of helpful analysis.

There are surgeons who tell you that clinical judgment is a sufficient guide to the safety of suture. For myself, I prefer to be guided, not to say controlled, by reference to the bacteriological suture standard. It is quite true that in the bulk of cases bacteriological findings and clinical judgment run obviously side by side, but sometimes they do not. Generally, however, when the bacteriologist reports that the smears are up to suture standard you find a characteristically healthy wound with suppuration stopped; also small, dark red, uniform and firm granulations; a regular and rapid ingrowth of epithelium; and a complete absence of all redness, swelling, and tenderness in the surrounding parts. The converse is not always true. The smears must of course always be made from the most dangerous situations, in and around exposed bone, tendinous or fascial structures, in all kinds of corners and recesses and from the skin edges. They should be spread as evenly as possible, the aim being to secure a layer of cells all contiguous and none superimposed. Such fields can practically always be found for examination where reasonable care has been taken. They should be stained with methylene blue, as other stains, notably gram, may give quite different results. Two consecutive favorable results with an interval of twenty-four hours between, suffice in the case of a simple flesh wound, but where there is a fracture present or any complication such as difficulty in closing the wound, I ask for three favorable reports and a culture showing the absence of streptococci or, in default of that, four or even five reports " up to suture standard ". There are of course those who cry out about the unscientific character of this method. On the other hand we who have used it are able to say " it works " and further I believe it furnishes a reliable index to the dose of infection present, and *that* rather than a knowledge of the nature of the infecting organism, is what seems to matter mainly in my experience.

Granted that this standard has been observed, it is amazing what liberties one may take. Flaps may be cut and turned over the wound, wide undermining of skin may be done, tissue grafts may be used — free or pedicled — to fill bone and other cavities, nerve suture may be practised, or redundant muscle excised when necessary — for instance, to facilitate suture of an amputation stump. I say these liberties may be taken. They should only be taken after careful consideration and after a corresponding tightening up of the suture standard. Each brings with it added risk of sepsis.

Other things that have to be considered are (1) the age of the wound — interference possible at 10 days might quite conceivably lead to utter failure at 40 days; (2) the size and conformation of the wound — if the suture standard be adhered to, there are few wounds that cannot be closed by one method or another, but there are some where the difficulty will be so great and the advantage gained so relatively small, that the operation is not justifiable; (3) multiplicity of wounds. It is not uncommon for one wound to reach the suture standard before others. Speaking generally, it is usually best to wait till all are ready before proceeding to suture. There are, however, certain exceptions :

*a*) The case of the wound in an awkward position, e. g., on the buttock or back when a man is compelled to lie on his back.

*b*) The wound which is so large that if sutured at the same time as the rest it will convert what ought to be a short and simple procedure into a long and serious operation.

*c*) The wound which entails a specially painful dressing, e. g., where nerve trunks are exposed. These I think should always be closed at the earliest possible moment.

*d*) The wound which by its position interferes with urgent orthopedic treatment.

The technique employed in wound suture is so simple as to require no set description. I will refer therefore only to certain isolated points. Cleaning of the skin is of primary importance. Attention to this point made more difference in my results than any other factor. I wash all round with neutral sodium oleate, the wound meanwhile being protected with a thick swab. The skin is then dried and the whole area washed with ether, dried in certain cases with alcohol, and the skin finally painted with iodine. All aseptic precautions being taken, the wound is closed with interrupted suture — silkworm gut is the best. I have carefully examined the comparative usefulness of silkworm gut, silk prepared in various ways, catgut, silver wire, and Mitchel's clips.

The guiding principle should be the restoration as near as may be to normal, with a minimum additional trauma. One can sometimes pass sutures so deep as not to enter the wound at all, but generally it is unnecessary to do so : however, there need be no hesitation in taking this step. In wounds not more than 12 days old, there is rarely any difficulty in closing the wounds. In older wounds it is often necessary to undermine the skin edges and it is advisable in such cases to resect the new cutaneous margin which is otherwise very apt to necrose. The wound being closed it is sometimes found that the edges are dead white from undue tension. Superficial incisions, parallel to the wound edge, which do not completely divide the skin, allow the skin to yield, and, at the same time, allow of a rapid escape of fluid from the tissues, and consequent diminution of swelling. Such " relief scarification " I have found very useful.

The last thing before applying the dressing, firm pressure is made with a roll of gauze to express all blood clot, and with dry dressing, plenty of wool, and a very firm bandage, the even pressure is maintained. The parts are placed in the position of greatest relaxation and when any doubt of success exists, are splinted, plaster of Paris being used if need be.

The dressing is left if possible untouched for seven days; after which time it is taken down and as a rule, every second stitch is removed. The remaining stitches are cut on the tenth day, though sometimes they have to be left much longer than this.

With regard to various methods of treatment, I desire to say very little. The whole subject is too full of fallacy and contention. Most of the cases to which I have referred had been treated before suture by the Carrel-Dakin method, but many other methods, including Flavine, hypertonic saline, Dichloramine T. in eucalyptol, soap, and B. I. P. P. had been used. If you ask my own personal opinion, I say without any hesitation that in the treatment of sepsis, the best results I have seen, and the best results I have obtained for myself, have been with the Carrel-Dakin method of treatment. The accompanying chart refers to comparable series of fifty cases each, all infected wounds, treated by various methods, and is intended to represent the relative speed with which wounds treated in the different ways reach the standard necessary for suture. Along the abscissa are marked the percentages, and along the ordinate the number of days of treatment. The uninterrupted line representing cases treated by the Carrel-Dakin method is seen to rise much more rapidly than the others, the maximum difference being seen about the twelfth day, when there were 40 o/o of the Carrel cases ready for suture, and only 14 to 18 o/o of those treated by the other methods.

Chart showing graphically relative speed with which infected wounds reach the Suture Standard under various modes of treatment.

In conclusion there are two points to which I wish to give special prominence. First, I am sure that the bacteriological examination of all wounds admitted to a base hospital, which are not obviously infected badly, will reveal many opportunities for delayed primary suture and will amply repay the surgeon. When I started this kind of work, I was astonished to find wounds capable of being easily and safely sutured, which at that stage of my experience seemed quite out of the question. In the second place, I would urge upon you the importance of strict adherence to the bacteriological control in all but the most exceptional cases. I believe it saves time in the end. I am certain that it saves the surgeon much anxiety, and I think that it provides a rational, reasonable and easily applied check upon the activities of the more ardent spirits amongst us, whose courage is apt to run away from time to time with their surgical judgment.

## Figures and Tables

**Figure f1:**